# Analysis of the Spectrum of *ACE2* Variation Suggests a Possible Influence of Rare and Common Variants on Susceptibility to COVID-19 and Severity of Outcome

**DOI:** 10.3389/fgene.2020.551220

**Published:** 2020-09-29

**Authors:** Anton E. Shikov, Yury A. Barbitoff, Andrey S. Glotov, Maria M. Danilova, Ziravard N. Tonyan, Yulia A. Nasykhova, Anastasia A. Mikhailova, Olesya N. Bespalova, Roman S. Kalinin, Azizahon M. Mirzorustamova, Igor Yu Kogan, Vladislav S. Baranov, Alexander N. Chernov, Dragana M. Pavlovich, Sergey V. Azarenko, Mikhail A. Fedyakov, Victoria V. Tsay, Yuri A. Eismont, Olga V. Romanova, Dmitry N. Hobotnikov, Dmitry A. Vologzhanin, Sergei V. Mosenko, Tatiana A. Ponomareva, Yana A. Talts, Anna U. Anisenkova, Dmitrii G. Lisovets, Andrey M. Sarana, Stanislav P. Urazov, Sergey G. Scherbak, Oleg S. Glotov

**Affiliations:** ^1^Genetics Laboratory, City Hospital No. 40, Saint Petersburg, Russia; ^2^Saint Petersburg State University, Saint Petersburg, Russia; ^3^Department of Genomic Medicine, D.O. Ott Research Institute of Obstetrics, Gynecology and Reproductology, Saint Petersburg, Russia; ^4^Bioinformatics Institute, Saint Petersburg, Russia

**Keywords:** COVID-19, *ACE2*, mutations, eQTL, gnomAD, whole-exome sequencing, allele frequency, Russia

## Abstract

**Objectives:**

In March 2020, the World Health Organization declared that an infectious respiratory disease caused by a new severe acute respiratory syndrome coronavirus 2 [SARS-CoV-2, causing coronavirus disease 2019 (COVID-19)] became a pandemic. In our study, we have analyzed a large publicly available dataset, the Genome Aggregation Database (gnomAD), as well as a cohort of 37 Russian patients with COVID-19 to assess the influence of different classes of genetic variants in the *angiotensin-converting enzyme-2* (*ACE2*) gene on the susceptibility to COVID-19 and the severity of disease outcome.

**Results:**

We demonstrate that the European populations slightly differ in alternative allele frequencies at the 2,754 variant sites in *ACE2* identified in the gnomAD database. We find that the Southern European population has a lower frequency of missense variants and slightly higher frequency of regulatory variants. However, we found no statistical support for the significance of these differences. We also show that the Russian population is similar to other European populations when comparing the frequencies of the *ACE2* variants. Evaluation of the effect of various classes of *ACE2* variants on COVID-19 outcome in a cohort of Russian patients showed that common missense and regulatory variants do not explain the differences in disease severity. At the same time, we find several rare *ACE2* variants (including rs146598386, rs73195521, rs755766792, and others) that are likely to affect the outcome of COVID-19. Our results demonstrate that the spectrum of genetic variants in *ACE2* may partially explain the differences in severity of the COVID-19 outcome.

## Introduction

A pneumonia of an unknown origin detected in late December 2019 in China turned out to be caused by a novel coronavirus, later named as severe acute respiratory syndrome coronavirus 2 (SARS-CoV-2), causing coronavirus disease 2019 (COVID-19) (Ludwig and Zarbock, 2020). Lately, despite the substantial effort of the Chinese government to halt the spread of this emerged infection, a disease that started from a local seafood market in China, it has become a world-scale threat. In March 2020, the World Health Organization (WHO) declared that COVID-19 became a pandemic. As a result of the COVID-19 pandemic, more than 15 million people have been infected, and more than 650,000 patients have died so far (as of July 28, 2020; source: Johns Hopkins University).

Patients with chronic diseases, such as diabetes, or elderly people, have the highest risk of developing severe symptoms or die from COVID-19 (Guan et al., 2020). The disease’s incubation period constitutes up to 14 days, with 3–7 days being the most common numbers (Wang et al., 2020). COVID-19 infection is characterized by multiple routes of transmission predominantly related to the respiratory tract. The dominant symptoms are cough and fever; however, other clinical manifestations are reported, including fatigue, sputum production, shortness of breath, sore throat, headache, and rarely diarrhea and vomiting (Poston et al., 2020). On average, adults and children experience flu-like symptoms or asymptomatic infection, despite some patients promptly developing acute respiratory distress syndrome (ARDS), capable of causing organ failure, impaired respiration, and even death (Guan et al., 2020). The mortality rate for patients in critical conditions could reach up to 49% (Wu and McGoogan, 2020).

Coronaviruses represent single positive-stranded RNA viruses with the largest genomes of all RNA viruses (Woo et al., 2009). These viruses gained their designation as a result of the crown-like form of viral particles composed of viral proteins (such as the spike protein). The comparative genomics study of different SARS-CoV-2 strains revealed two major types of the virus, the L-type (≈70% of cases) and the S-type (≈30% of cases). L-type strains surpass S-type ones in terms of aggressiveness and infectiousness (Tang et al., 2020). SARS-CoV-2 mostly infects the lungs, heart, liver, kidney, small intestine, and testes, where there is a high expression of its target receptor – angiotensin-converting enzyme-2 (ACE2) (Anguiano et al., 2017). The virus binds to ACE2 receptors in humans *via* its surface proteins (Fehr and Perlman, 2015). When the S (spike) protein on the surface of SARS-CoV-2 binds to ACE2, a viral particle fuses with the host cell membrane utilizing serine protease transmembrane protease, serine 2 (TMPRSS2) (Hoffmann et al., 2020; Zhou et al., 2020).

The interaction of the S-protein with ACE2 indicates that mutations and single-nucleotide polymorphic substitutions (SNPs) in the *ACE2* gene can affect the affinity and specificity of S-protein binding to ACE2, being the molecular basis for individual variation in susceptibility to the infection. A simple hypothesis could link the severity of infection with variation in the sequence of the *ACE2* receptor gene. When comparing world populations based on ChinaMAP and 1KGP (The 1000 Genomes Project) data, no S-protein binding-resistant *ACE2* mutants were found (Cao et al., 2020). However, the prevalence of expression quantitative trait locus (eQTL) variants in the East Asian population was reported (Chen et al., 2020). Nonetheless, in both studies, most variants assessed were intronic, indicating their unclear association with the functionality of the ACE2 protein. Furthermore, the first study (Cao et al., 2020) did not discuss the *ACE2* gene polymorphism in different European subpopulations. Recent whole-exome sequencing (WES)-based studies in such countries as Italy also aimed at the identification of the genetic COVID-19 susceptibility factors, with some missense *ACE2* variants shown to have a protective role (Benetti et al., 2020). The comprehensive study of the *ACE2* variations focused on the possible protective pathogenic mutations revealed that missense mutations affecting protein interaction with S-protein exhibit overall low frequency due to the absence of natural selection with no discernable differences between populations (Stawiski et al., 2020).

In the present article, we attempted to elaborate on the spectrum of variants in *ACE2* in different European populations and its possible relationship to COVID-19 severity. We also investigated the frequencies of the identified variants in Russia and evaluated the possible role of these variants using a cohort of 37 Russian patients with COVID-19.

## Materials and Methods

### Data Acquisition From Genome Aggregation Database

To analyze variants in the *ACE2* gene, we first retrieved variant site data from the largest openly available variant compendium, the Genome Aggregation Database (gnomAD) v2.1.1 (Karczewski et al., 2020). Both exomes and genomes in X chromosome sites were used. We then selected variants inside the *ACE2* region defined in GENCODE v19. Allele counts and allele numbers for SNPs called in both genome and exome datasets were used to estimate total frequencies for each population (the resulting table is available as Supplementary Table S1).

### Analysis of the *ACE2* Expression Quantitative Trait Loci

Significant *cis-*eQTLs for the *ACE2* gene, contributing to *ACE2* expression, were described in a recent work by Chen et al. (2020). To expand the list of variants, we used the GTEx v. 7 (The Genotype-Tissue Expression) consortium dataset (Lonsdale et al., 2013) and extracted all significant (adjusted *p* < 0.05) single-tissue eQTLs belonging to *ACE2* (*ENSG00000130234*) (Supplementary Table S2). For multi-tissue eQTLs, we considered tissues with the lowest *p*-value. We then combined Chen et al.’s (2020) and GTEx-based eQTL sets and annotated these variants with allele frequency data from gnomAD (results are available as Supplementary Table S3).

### Whole-Exome Sequencing Data

An updated version of the Russian exome variant dataset (Barbitoff et al., 2019) was used in this study. A total of 665 previously undescribed samples were added to the dataset, with most of the individuals being of European ethnicity (self-reported) and residents of the northwestern region of Russia. All participants (1,359) were not COVID-19 patients; thus, they can be considered a population control.

For additional samples (not described in Barbitoff et al., 2019), library preparation and sequencing were performed as follows: DNA samples from the blood of all patients were isolated by phenol extraction. DNA concentration was determined using Quantus Fluorometer^TM^ and QuantiFluor^®^ dsDNA System (Promega Corporation, Madison, WI, United States). DNA integrity was verified using electrophoresis in 0.6% agarose gel in sodium borate (SB) buffer. Exome DNA libraries were prepared from 100 ng DNA sequenced with both WES kits (Roche SeqCap EZ MedExome and Illumina TruSeq Exome Sample Preparation kit) and clinical exome panel (CES) [Roche Inherited Disease Panel (IDP) v2], following the manufacturer’s instructions. Libraries were sequenced on Illumina MiSeq, HiSeq 2500, and HiSeq 4000. Quality control was executed with HiSeq/MiSeq Control Software v3.3 and Real-Time Analysis v2.7.3. Base-calling was performed with the Illumina bcl2fastq (v2.17.1.14) package.

Bioinformatic analysis of the exome sequencing data was performed as described in Barbitoff et al. (2019). Only variants inside the *ACE2* region (chrX:15,579,156-15,620,271) were considered in this analysis.

### COVID-19 Patient Groups

Thirty-seven patients with a diagnosis of SARS-CoV-2 and 21 randomly chosen donors were included in this study. The main parameters of the participants are given in Supplementary Table S4. Diagnosis of SARS-CoV-2 and the COVID-19 severity were confirmed following the recommendations of the Ministry of Health of Russian Federation^1^ (the criteria are given in Supplementary Table S5). All the patients and donors were residents of Northwest Russia. The study was performed in accordance with the Declaration of Helsinki. A signed written informed consent was obtained from all participants. Ethics approval was obtained from the ethics committee of the review board of City Hospital No. 40.

Blood levels of major inflammation-related molecules were analyzed. The C-reactive protein level was measured on automated biochemistry analyzer Architect c8000 (Abbott). Ferritin and interleukin-6 levels were assessed *via* Cobas e411 modular analyzer (Roche). The lymphocyte count was evaluated *via* Automated Hematology System Sysmex XN-1000^TM^ (Sysmex Inc., United States).

### Long-Range Amplification and Sequencing of *ACE2*

Genomic DNA was isolated from the whole venous blood samples using salt out extraction protocol. DNA concentration was determined using fluorometric quantification by Qubit 2.0 (Thermo Fisher Scientific Inc.).

The *ACE2* gene amplification was performed by long-range polymerase chain reaction (LR-PCR). The *ACE2* gene sequence was amplified as six overlapping fragments, ranging between 7,084 and 9,478 bp, which covered the entire gene (Table 1). Primers’ sequences were designed with “Oligo 6” (United States) and “NCBI BLAST” (United States) software. PCR was performed with the LongAmp^®^ Taq 2X Master Mix (New England Biolabs Inc., United States).

**TABLE 1:** Primers of the *ACE2* gene fragments used for the amplification.

**Primer design, 5′—>3′**	**Tm, °C**	**Fragment size, bp**
F1: GTTTCACCATGTTGCCCAGGCTGGTCTTGAACTCTT	68	9,053
R1: CACCCTTGGCTCCCATATGCTGGCTCAATTTCCAGA		
F2: CAAGTACCTGGGCATATTTAGAAAGTAACCAAGGCA	68	9,478
R2: GGAAGAACATAAGGGAAAGAAAGAGAGAAAGCACAA		
F3: TTTAAAAATAAACATAGGCTGTTACTCTGCACAGTG	68	9,222
R3: ACAAACTCTCCTTCACTTATTTCTCATTCTAGCCTA		
F4: ACCCAAGCTGTGAGAACAGCAGGATCAAATACAG	68	9,187
R4: CATAAAATGGCAGCTGTCACCATAGCAGAGAAAG		
F5: ACAGCTGAGAATGTTTCTTTCTCTATAGTACCTGCT	68	9,000
R5: AAATATGCAGAACATTGTTTATAGCCTGCTAACATT		
F6: ATGTGCATATGTATATCTCATTTCTTAGTATACAAA	60	7,084
R6: TTATATAGTCAAGTCTACATTTGCATTTTTCAGC		

The efficiency of the long-range PCR amplification was verified with 0.8% agarose gel and further visualization through ethidium bromide staining. The amplicon concentration was quantified with Qubit software (Thermo Fisher Scientific Inc.) following the manufacturer’s instructions. The six LR-PCR fragments were pooled in equimolar amount for each DNA sample and then sheared with Bioruptor^®^ Sonication System (Diagenode), following the manufacturer’s instructions to obtain the 150-bp mean fragment size.

Libraries were constructed with the Ion Plus Fragment Library Kit (Thermo Fisher Scientific Inc.) according to the manufacturer’s instructions. Validation and quantification of the libraries were performed with the 2200 TapeStation Instrument (Agilent Technologies). An automated system Ion Chef Instrument was used to prepare libraries and load the Ion 540 Chip (Thermo Fisher Scientific Inc.). Sequencing was performed with the Ion Torrent S5 System machine (Thermo Fisher Scientific Inc.).

### Analysis of Ins/Del Polymorphic Variant of *ACE1*

To detect the insertion/deletion (I/D) polymorphic variant of *ACE1* (defined as four consecutive small insertion/deletion variants in the *Alu* sequence of intron 16 of *ACE1*), the rs1799752 genotype was assessed using a standard PCR protocol. PCR was used with the following primer design: 5′-CTGGAGACCACTCCCATCCTTTCT, 3′-GATGTGGCCATCACATTCGTCAGAT. PCR products were analyzed in 6% polyacrylamide gel and visualized with ethidium bromide staining. The association between rs1799752 genotype and COVID-19 severity was tested using a chi-square test.

### Semiconductor Sequencing Data Analysis

Semiconductor sequencing data were processed using an in-house pipeline based on the bwa mem v. 0.7.15-r1140 alignment (Li and Durbin, 2011), GATK v. 3.5 read pre-processing (indel realignment base recalibration), DeepVariant v. 0.10.0 variant caller (Poplin et al., 2018), and cohort genotyping using GLnexus (Lin et al., 2018). The resulting variant calls were filtered to exclude small indels in homopolymeric sequence regions as common sequencing errors. Filtered variants were annotated using SnpEff and SnpSift v.4.2 (Cingolani et al., 2012).

### Statistical Analysis

Data visualization and statistical analysis were performed in R v.3.6.2 using the ggplot2 v.3.3.0 (Wickham, 2016) and ggsci v2.9 packages. To statistically test whether eQTL allele frequencies differ across populations, we applied a fitted linear regression model to predict allele frequencies based on variant rsID and the population. The significance was then evaluated using the *t*-based *p*-value describing the significance of a regression coefficient for a particular population. To evaluate the significance of the differences between populations when analyzing missense variants’ frequency, we randomly selected 1,000 sets of SNPs located on the X chromosome with the size of the set and global allele frequency distribution matching the ones for all missense or deleterious missense variants in the *ACE2* gene. The empirical *p*-values were then calculated as the fraction of sampling replicates in which the simulated total frequency of SNPs in the set was less than the observed total frequency of all missense or deleterious missense variants.

Allele counts distribution uniformity for each variant in the cohort of COVID-19 patients was tested using the chi-squared test to compare allele counts and sample sizes. A comparison of blood C-reactive protein concentration and other measurements between patient groups was performed using the Wilcoxon–Mann–Whitney rank-sum test.

## Results

### Differences in *ACE2* Variants’ Frequencies Between Genome Aggregation Database Populations

The first goal of our analysis was to investigate the differences in the spectrum of *ACE2* genetic variants in gnomAD (Karczewski et al., 2020). We focused mostly on the European subpopulations: Finnish European (FIN) and non-Finnish European (NFE), including Northwestern European (NWE), Bulgarian (BGR), Estonian (EST), Swedish (SWE), Southern European (SEU), and Other non-Finnish European (ONF). As a reference for comparison, we also included the African (AFR), East Asian (EAS), and South Asian (SAS) populations. Overall, 620 variant sites in the *ACE2* gene were present in the gnomAD exome dataset and 2,232 in the genome dataset. In total, we selected 2,754 variant sites from both datasets (Supplementary Table S1). Expectedly, the vast majority of variants (2,383) were non-coding, 122 were synonymous or splice region variants (not affecting donor or acceptor sites), and 249 variants were non-synonymous, with eight high-impact variants (nonsense, frameshift, or canonical splice site variants) (Supplementary Figure S1). Out of the non-coding variants, most were intron (1,804), downstream (226), and upstream (247) variants (Supplementary Figure S2).

**FIGURE 1:**
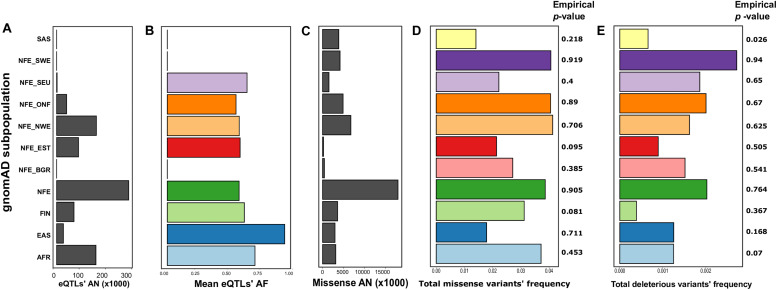
The differences between populations based on the Genome Aggregation Database (gnomAD) data (Karczewski et al., 2020). Shown are mean allele numbers (ANs) **(A,C)** and allele frequencies (AFs) **(B,D,E)** for selected classes of variants. For eQTLs **(B)**, mean AFs are shown; for missense sites **(D)**, and deleterious missense variants **(E)**, total AF is shown. Empirical *p*-values were derived from 1,000 random simulations of single-nucleotide polymorphic substitution (SNP) sets from the ChrX region. AFR, African; EAS, East Asian; FIN, Finnish; NFE, non-Finnish European; NFE_BGR, Bulgarian; NFE_EST, Estonian; NFE_NWE, Northwestern European; NFE_ONF, other non-Finnish European; NFE_SEU, South European; NFE_ SWE, Swedish; SAS, South Asian.

We then went on to specifically assess differences in frequencies of two essential groups of SNPs: variants, significantly affecting expression level (eQTLs), and missense mutations. For eQTL analysis, we combined the sets of significant eQTLs for *ACE2* described in a recent article by Chen et al. (2020) with single-tissue eQTL data from the GTEx v.7 data (Lonsdale et al., 2013). In total, we obtained 35 variants, 33 of which were located on intron regions, while two represented downstream sites (Supplementary Table S3). Interestingly, *ACE2* eQTLs most significantly affected gene expression in the nervous system, with 11 variants influencing gene expression in the brain and 24 variants in the Nerve-Tibial (Supplementary Figure S3). We next calculated the mean allele frequency for all eQTLs for each population. Interestingly, we found that in the SEU population, the mean frequency was the highest of all European populations (0.65 vs. 0.58 for combined non-Finnish European) (Supplementary Table S6 and Figures 1A,B). The difference was also statistically significant when applying a linear regression-based test (*p*-value for the SEU = 0.0126, see “Materials and Methods”). However, this result could, at least in part, be explained by the small number of genome samples for the SEU population in gnomAD (Figure 1A).

To compare the abundance of missense mutations, we calculated the total frequency of missense variants for each analyzed population by summing the frequencies across all missense sites. Such an approach assumes that missense variants rarely co-occur in the same individual; this assumption is justified given that almost all of the missense variants in the *ACE2* gene are rare in gnomAD. Notably, the total frequency of missense variants in the SEU population was the lowest (0.022), being more similar to the frequency in the East Asian population and Estonians (Supplementary Table S6 and Figures 1C,D). The low frequency of missense variants in the SEU population suggests that missense variants might play a protective role against COVID-19 infection (a similar hypothesis has been made by Benetti et al. (2020). Surprisingly, the differences between populations are reversed when considering missense variants predicted as deleterious by PROVEAN (Choi and Chan, 2015), SIFT (Ng and Henikoff, 2001), FATHMM (Shihab et al., 2013), and PolyPhen2 (Adzhubei et al., 2010; Supplementary Table S7 and Figure 1E).

To validate that the differences between subpopulations are not explained by sample sizes or other technical confounders, we conducted a comparison of the missense and deleterious mutations’ frequencies with randomly selected ChrX-located sites (see “Materials and Methods”). Unfortunately, this analysis showed lack of strong statistical support of the observed differences, as random SNP sets matching all missense or deleterious missense variant groups frequently had comparable differences in allele frequency [except for the deleterious mutations in the SAS population (Figure 1)]. This observation does not allow us to make confident conclusions about the functional impact of these mutations on the COVID-19 susceptibility/severity of the disease.

### The Spectrum of Variants in the Russian Population

To characterize the spectrum of variants in the *ACE2* gene in the Russian population, we analyzed an updated version of the WES dataset published previously (Barbitoff et al., 2019) (see “Materials and Methods”). The raw dataset was composed of 1,359 samples. We filtered out samples based on the mean coverage of target regions (exons of the *ACE2* gene, Supplementary Table S8) to ensure that we can accurately estimate the allele frequencies. We excluded samples with a mean coverage less than 20× across *ACE2* exons. Only 550 samples passed such filtering (540 WES samples and 10 CES samples). Ninety-eight percent of all considered sites were covered with at least 10× mean depth, and 47% of the sites were covered at least 50×.

We identified a total of 82 *ACE2* variants in the Russian exome dataset (Supplementary Table S9); most of them were annotated as intronic (68) and upstream gene variants (11). As exome panels mainly encompass exons, intron calls are expected to contain high amounts of false positives. Hence, we applied the filtering of loci based on site depth, retaining only variants with at least 10× mean coverage across all samples (Supplementary Figure S4). This procedure has dramatically reduced the number of high-quality variant sites, with only eight variants remaining (Table 2); three of these were intron variants present only as a single heterozygous call. We next considered the remaining five coding variants. rs41303171 (p.Asn720Asp) missense variant and the rs35803318 synonymous SNP are rather common variants for the European population (Cao et al., 2020), as well as a splice site rs2285666 variant. Finally, rs113691336 and rs971249 are significant eQTLs in the Nerve-Tibial tissue (*p*-values 7.3 × 10^–9^ and 1 × 10^–8^, respectively).

**TABLE 2:** Variants detected in Russian exomes after the filtering procedures.

**Position**	**rsID**	**Ref**	**Alt**	**Effect**	**Pathogeny prediction**	**Protein**	**Homozygotes**	**Heterozygotes**	**AF**
15582209	rs35803318	C	T	SYNON	Pathogenic	p.Val749Val	10	13	0.031
15582298	rs41303171	T	C	MISS	Pathogenic	p.Asn720Asp	2	15	0.016
15596143	rs113691336	C	CATAAG	INTRN	–	–	232	82	0.609
15606024	–	T	TTC	INTRN	–	–	1	1084	0.001
15606028	–	A	ATTGT	INTRN	–	–	1	1084	0.001
15606029	–	A	ATTACTTT	INTRN			1	1084	0.001
15607650	rs971249	T	C	INTRN	Benign	–	278	142	0.671
15610348	rs2285666	C	T	SPLIR	Benign	–	66	89	0.205

*Annotations of called variants found in the Russian population. Position of variants is provided according to hg19 coordinates. The effect was delineated via SnpEff (Cingolani et al., 2012); pathogeny prediction was obtained from FATHMM (Shihab et al., 2013). The numbers of homozygotes, heterozygotes, and allele frequency (AF) were calculated for the alternative allele.*

Unfortunately, the limited number of identified variant sites in *ACE2* in our dataset is insufficient for comprehensive analysis. However, it can be seen that Russia falls in close proximity to other Europeans based on allele frequencies (Supplementary Table S10). In order to assess the statistical significance of the differences between Russia and other populations for different variants, we performed Fisher’s exact test comparisons using allele numbers and allele count for each variant with subsequent false discovery rate (FDR) adjustment (Supplementary Table S11). According to the adjusted *p*-value, highly significant differences were found between Russian and East Asian and African populations. The rest of the comparisons with European populations did not reveal substantial statistical differences for almost all variants except for rs35803318 (*p*-value = 0.011 for Finnish, *p*-value = 0.048 for Northwestern European, and *p*-value = 0.028 for South European populations).

Overall, we conclude that the frequencies of the *ACE2* variants in the population of Northwest Russia do not substantially differ from the ones observed in the other European populations. Hence, similar effects of the *ACE2* variation on the disease outcome might be observed in Russia.

### Analysis of the Genetic Variants’ Spectrum in a Cohort of Russian COVID-19 Patients

To assess whether the observed differences in functional *ACE2* variants’ frequencies affect COVID-19 susceptibility and/or severity, we analyzed a cohort of 37 patients with either mild or severe form of COVID-19 (see “Materials and Methods”). To perform a comprehensive analysis of genetic variants in the *ACE2* gene, we utilized long-range amplification of the whole gene sequence followed by semiconductor sequencing. A sample of 21 donors from the matching population was used as a control group.

**FIGURE 2:**
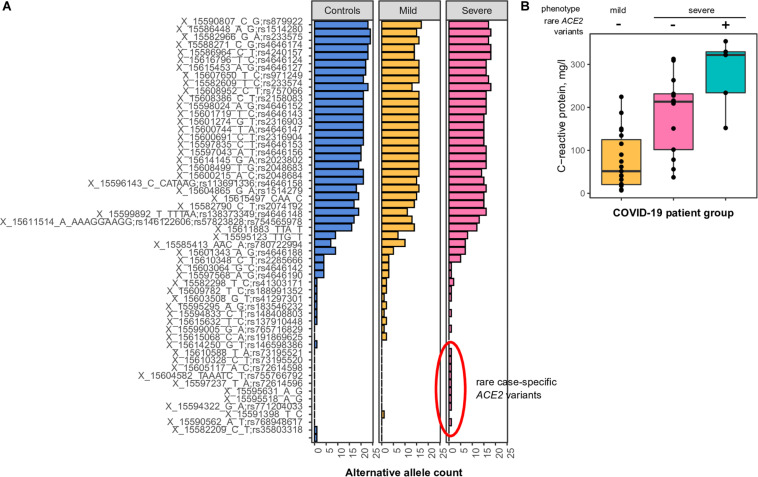
**(A)** Common and rare variants of the *angiotensin-converting enzyme-2* (*ACE2*) gene in different coronavirus disease 2019 (COVID-19) patients. Alternative allele counts for 54 single-nucleotide polymorphic substitutions (SNPs) and indels among patients with mild and severe forms of the COVID-19 infection. **(B)** Mean concentration of C-reactive protein in the blood of COVID-19 patients with either mild or severe type of disease. The difference between CRP concentrations in patients with severe COVID-19 carrying or not carrying rare *ACE2* haplotypes is significant in the Wilcoxon-Mann-Whitney rank-sum test (*p* < 0.05).

We discovered a total of 54 SNPs and large indels (Figure 2A). Statistical comparison of the individual variants’ frequencies between patients with mild and severe COVID-19 did not reveal any significant differences. We then asked whether the total number of eQTL alleles is different between the two groups. Unfortunately, no difference was found in such an analysis (333 alleles in patients with mild COVID-19 compared to 339 in patients with severe COVID-19). Given these results, we conclude that, while certain differences in *ACE2* eQTL frequencies across populations exist (Figure 1A), these differences have either no or very little effect on the COVID-19 susceptibility and severity.

While analyzing the spectrum of discovered variants, we observed an unusually high number of rare singleton variants (i.e., variants observed only once across all patients and control subjects) in patients with severe COVID-19 (Figure 2A, nine variants compared to one variant in patients with mild disease manifestation, chi-square *p*-value = 0.018). These differences were less pronounced when analyzing the number of patients carrying such rare variants (five patients with severe COVID-19, one patient with mild disease form), as some of the identified rare variants are seemingly linked in rare haplotypes. We then asked whether these rare variants do indeed contribute to disease severity. To further validate our findings, we compared clinical features of the patients carrying the identified rare alleles to other patients with either severe or mild COVID-19 manifestation. We found a significant upshift in the plasma C-reactive protein levels in rare allele carriers compared to other study participants (Figure 2B, Wilcoxon rank-sum test *p*-value = 0.035), while no relationship to patients’ sex or age was observed. These results indicate that patients carrying the rare *ACE2* haplotypes tend to have a more active inflammatory response to SARS-CoV-2, which explains a certain proportion of the variation in disease severity.

Finally, we investigated whether a common I/D polymorphism of the *ACE1* gene can act as a modifier of COVID-19 severity in patients harboring rare and common *ACE2* alleles. A recent work by Gemmati et al. (2020) presented a hypothesis that the deletion allele in the intron 16 of *ACE1*, associated with higher *ACE1* expression, may alter ACE1/ACE2 balance and contribute to disease severity. We assessed the I/D genotype of the patients by PCR-based analysis of the allele combination at the rs1799752 locus. We found no relationship between the rs1799752 genotype and COVID-19 severity in our patients, and no association between the *ACE2* variation and the rs1799752 genotype was observed in our sample (data not shown).

## Discussion

Angiotensin-converting enzyme-2 acts as a receptor for SARS-CoV and SARS-CoV-2 by attaching to receptor-binding motif (RBM) in the receptor-binding domain (RBD) of the Spike protein on SARS-CoV particle surface (Li et al., 2003, 2005). The normal functions of ACE2 include the control of blood pressure and regulation of heart and kidney functions (Anguiano et al., 2017). It has been demonstrated that the specificity of interaction between S-protein and ACE2 is a major determinant of the host tropism and host range of SARS-CoV-2 (Luan et al., 2020). A bioinformatic assay on the ACE2 structure in diverse mammals revealed that pets (dog and cat), pangolin, and Circetidae mammals possess the most key residues accounting for interaction with SARS-CoV-2, thus providing support for the zootomic origin of COVID-19 (Luan et al., 2020). Recent advances in research of ACE2 based on cryo-electron microscopy revealed the high-resolution structure of full-length ACE2 (Yan et al., 2020). Molecular docking of the S-protein trimer onto the ACE2 dimer supports the idea that two S-protein trimers bind to an ACE2 dimer simultaneously. It was also clearly shown that RBD recognition by the extracellular peptidase domain of ACE2 is predominantly enabled through polar residues. This information could provide the basis for the further rational design of molecules that bind to ACE2 or the Spike protein in order to prevent viral interaction with host cells and repress infection. Given this crucial role of ACE2 in the interaction with SARS-CoV-2 interactions, it can be proposed that the genetic variation in the *ACE2* gene is likely to contribute to the susceptibility and severity of the disease. This hypothesis is now widely discussed in the literature and is being extensively investigated (Benetti et al., 2020).

Missense mutations in *ACE2* may perturb the receptor–ligand interactions. No S-protein binding-resistant ACE2 mutations are present in human populations; however, low-frequency missense variants could also affect the severity of infection (Cao et al., 2020). In our study, we showed that the frequencies of missense variants in the *ACE2* gene vary between populations, indicating a possible link between low missense variants frequency and COVID-19 manifestations. If a missense mutation affects the receptor function of ACE2, the higher frequency of missense allele carriers in some populations could result in a lower infection rate. The lower carrier frequency of missense variants, in turn, might increase susceptibility in populations like SEU. Indeed, some seemingly protective missense variants in *ACE2* found in a recent Italian (part of the SEU group) study showed lower allele frequencies compared to the NFE population in general (Benetti et al., 2020). While the common missense variants in *ACE2* might not affect host–virus interaction, they might have an indirect impact on COVID-19 susceptibility and disease outcome. For instance, putative ACE2 mutations can increase the oxidative stress rate, thus severing the outcome of the disease (Devaux et al., 2020). However, the absence of statistical differences between random SNP replicates may indicate that the nominal distinction between the populations simply reflects the insufficient representation of particular populations.

We find that the frequencies of regulatory variants affecting *ACE2* expression also differ across populations. While no effect of these variants on the *ACE2* expression in the lungs can be found, the effects of *cis-*eQTLs on ACE2 function in different brain tissues may have a link to neurological complications in patients with COVID19 (Strafella et al., 2020). We can also hypothesize that even slightly increased expression of *ACE2* may result in more molecules of the receptor on the cell surface, which in turn might increase the susceptibility to COVID-19. The same mechanism might hold true for the rare *ACE2* haplotypes, which were found to be overrepresented in Russian patients with the severe form of COVID-19 (Figure 2). However, it is also likely that the effect of these variants on the phenotype is also not related to the receptor function of ACE2.

*Angiotensin-converting enzyme-2* variants are associated with a wide variety of traits. It was shown that some variants in *ACE2* in Uygyrs with type 2 diabetes mellitus are associated with cardiovascular risk (Liu et al., 2018). These variants, such as rs1978124, rs2048683, rs233575, rs4240157, rs4646156, and rs879922, are significant *cis-*eQTLs for *ACE2* (Supplementary Table S3). The researchers from the Department of Epidemiology, Biostatistics, and Occupational Health, McGill University (Montréal, QC, Canada) found negative and positive associations of minor alleles of rs2074192 and rs233575 with systolic and diastolic pressure in French Canadians and European populations (*n* = 555) (Malard et al., 2013). The *A* allele of the rs2285666 variant was significantly associated with a risk of death from cardiovascular disease in women carrying two copies of the *ACE2* gene (Vangjeli et al., 2011). However, no association with the susceptibility and severity of coronavirus infection was reported (Chiu et al., 2004). The rs2285666 variant was significantly associated with a decrease in high-density lipoprotein (HDL) cholesterol (<1.0 mmol/L) in patients with essential hypertension (Pan et al., 2018). Another research showed that the *C* allele of rs2285666 was associated with a 2.82-fold risk of relapse in men with a history of stroke hypertension compared with patients without a history of hypertension but with the *T* allele (Wu et al., 2018).

In contrast to these data, Zhang et al. (2018) showed that the T/C genotype rs2285666 was significantly higher in the group of normotensive individuals compared with the group of hypertensive patients [32.0 vs. 44.8%, *p* < 0.001, odds ratio (OR) = 0.572, 95% CI 0.428–0.765]. These results indicate that the T/C rs2285666 genotype has a reduced risk of developing essential hypertension and is a factor in genetic resistance to its development (Zhang et al., 2018). These studies confirm the association of SNPs in the *ACE2* gene and the functionality of the ACE2 protein. Hence, it may be hypothesized that these and other variants (including the rare ones identified in this study), while not having a direct role in the interaction of the *ACE2* with virus particles, may play a role in COVID-19 pathology indirectly, affecting important normal functions of the protein.

It has been proposed recently that the balance between ACE1/ACE2 function is crucial for COVID-19 disease progression (Gemmati et al., 2020). Normally, ACE1 and ACE2 proteins are antagonistic enzymes and major components of the renin–angiotensin system (RAS) that regulates vascular function. A functional balance between ACE1/ACE2 defines the activity of the RAS, and increased expression of *ACE1* accompanied by downregulation of ACE2 may lead to severe ARDS. A deletion allele at the polymorphic locus in the intron 16 of *ACE1* has been shown to elevate the expression of *ACE1*, thus potentially contributing to the severity of COVID-19 (Gemmati et al., 2020). In our study, we tested the relationship between the I/D polymorphism of *ACE1* and COVID-19 severity and found no evidence for such a link. The results obtained could also be explained by the absence of the actual influence of the *ACE1* polymorphisms on gene expression, which is supported by other studies (Johnson et al., 2009). Nevertheless, potential physiological consequences of the alterations in the ACE1/ACE2 balance provide yet another plausible hypothesis that might explain the indirect effects of *ACE2* variants on SARS-CoV-2 infection.

## Conclusion

Overall, in this study, we examined the spectrum of genetic variants in the *ACE2* gene across human populations and revealed specific differences, particularly in missense variants and *cis-*eQTLs. However, the statistical analyses revealed that the distinctions observed are likely to be explained *via* the populational structure and the heterogeneity of the gnomAD data. Moreover, data obtained using a pilot cohort of Russian COVID-19 patients demonstrate that the differences in missense and eQTL allele frequencies do not explain the variability in the severity of the disease and have only a slight – if any – contribution to the phenotype. At the same time, we identified that rare *ACE2* haplotypes (not having any reported functional effect) are likely to predispose to severe COVID-19 infection and may account for certain (though not the majority of) severe COVID-19 cases.

## Data Availability Statement

The datasets presented in this study can be found in online repositories. Main data files and code are available in the GitHub repository: https://github.com/anton-shikov/ACE2.

## Ethics Statement

The studies involving human participants were reviewed and approved by the Review Board of City Hospital No. 40, Saint Petersburg, Russia. The patients/participants provided their written informed consent to participate in this study.

## Author Contributions

AES contributed to the conceptualization, data curation, methodology, and writing the original draft. YB contributed to the writing and editing, data curation, investigation, and methodology. AG contributed to the conceptualization, data curation, methodology, resources, and supervision. MD, ZT, AAM, RK, AMM, DP, SA, VT, YE, OR, DH, DV, SM, TP, YT, and AA contributed to the laboratory investigations. YN contributed to the writing and editing and laboratory investigations. OB, IK, DL, AMS, and SU contributed to the resources. VB contributed to the supervision. AC and MF contributed to the investigation and writing the original draft. SS contributed to resources and the supervision. OG contributed to the conceptualization, project administration, and supervision. All authors contributed to the article and approved the submitted version.

## Conflict of Interest

The authors declare that the research was conducted in the absence of any commercial or financial relationships that could be construed as a potential conflict of interest.

## Footnotes

1 http://edu.rosminzdrav.ru/fileadmin/user_upload/specialists/COVID-19/MR_COVID-19_v7.pdf
