# Supplementary material for: Analysis of the Spectrum of ACE2 Variation Suggests a Possible Influence of Rare and Common Variants on Susceptibility to COVID-19 and Severity of Outcome
Source: Front Genet. 2020 Sep 29;11:551220. doi: 10.3389/fgene.2020.551220 (PMC7550667; doi:10.3389/fgene.2020.551220)
Supplement: Supplementary file 1 [file Data_Sheet_1.PDF]

Supplementary Material

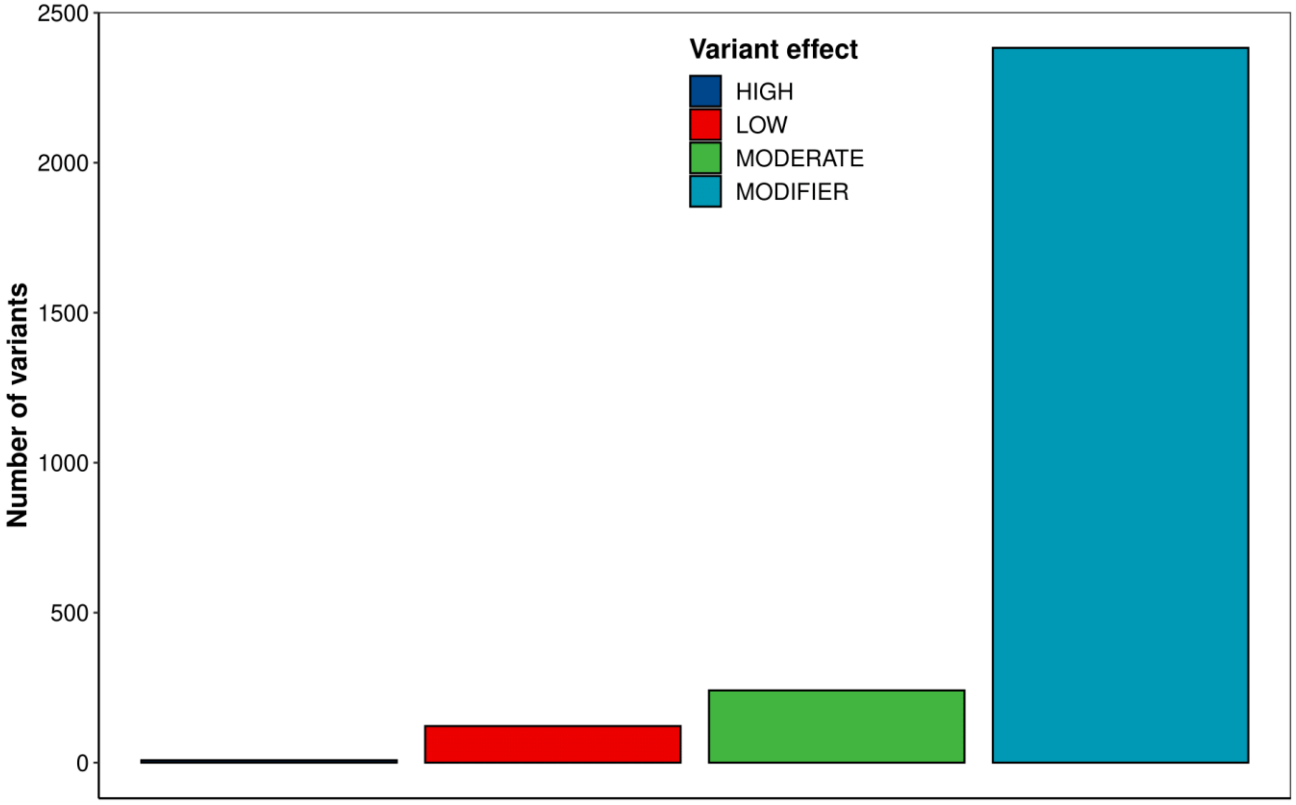

Supplementary Figure S1. Distribution of effect types for 2754 SNPs extracted from gnomAD v 2.1.1 related to the *ACE2* gene.

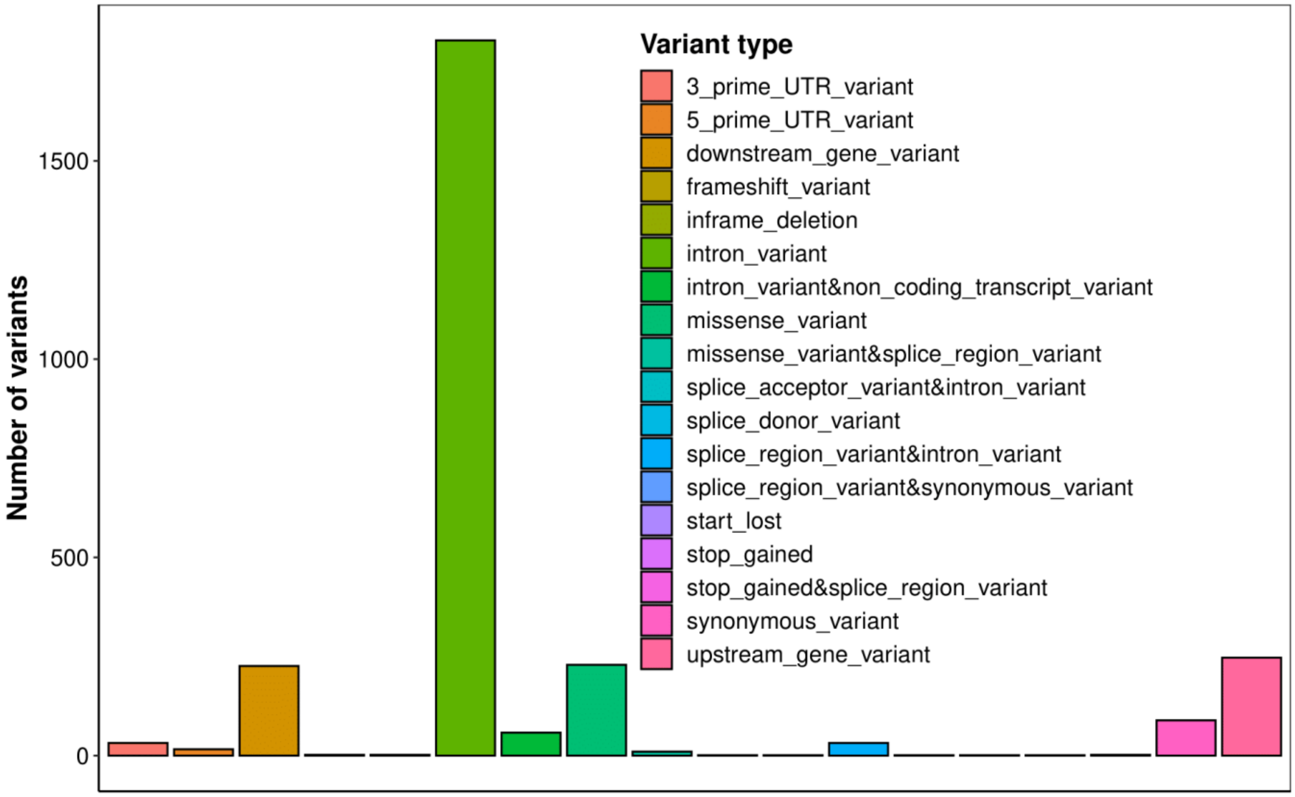

Supplementary Figure S2. A distribution of variant types for 2754 SNPs extracted from gnomAD v2.1.1 related to the *ACE2* gene.

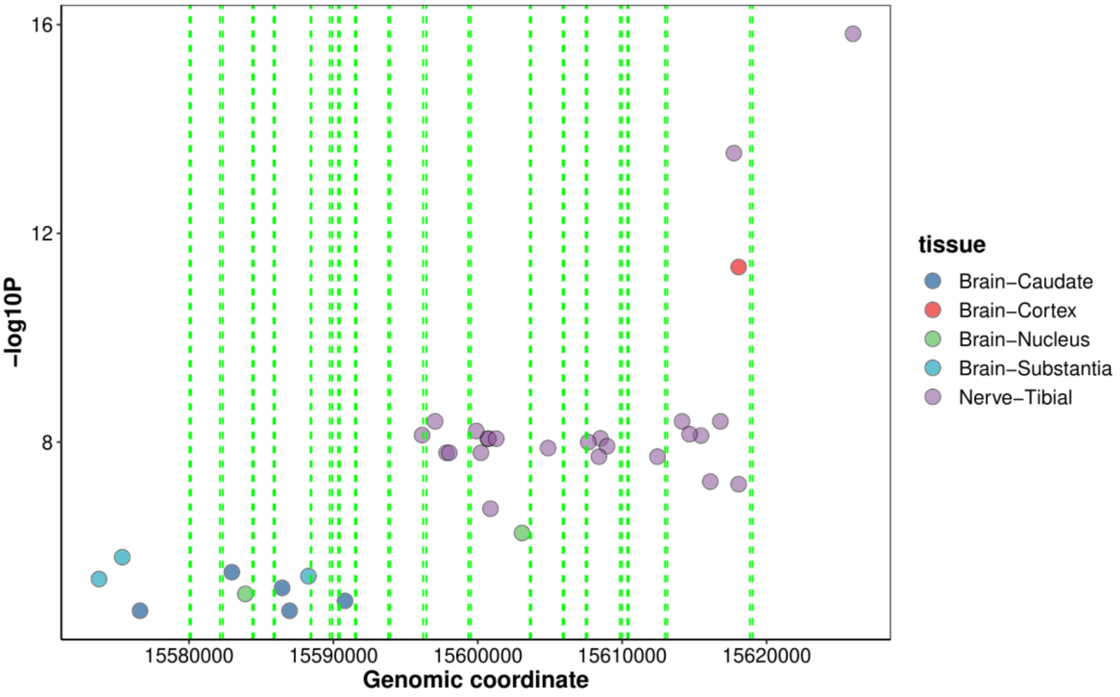

Supplementary Figure S3. The location on the genome and of significant eQTL variants studied in the article. The green dashed lines mark the beginning and end coordinates of the *ACE2* exons.

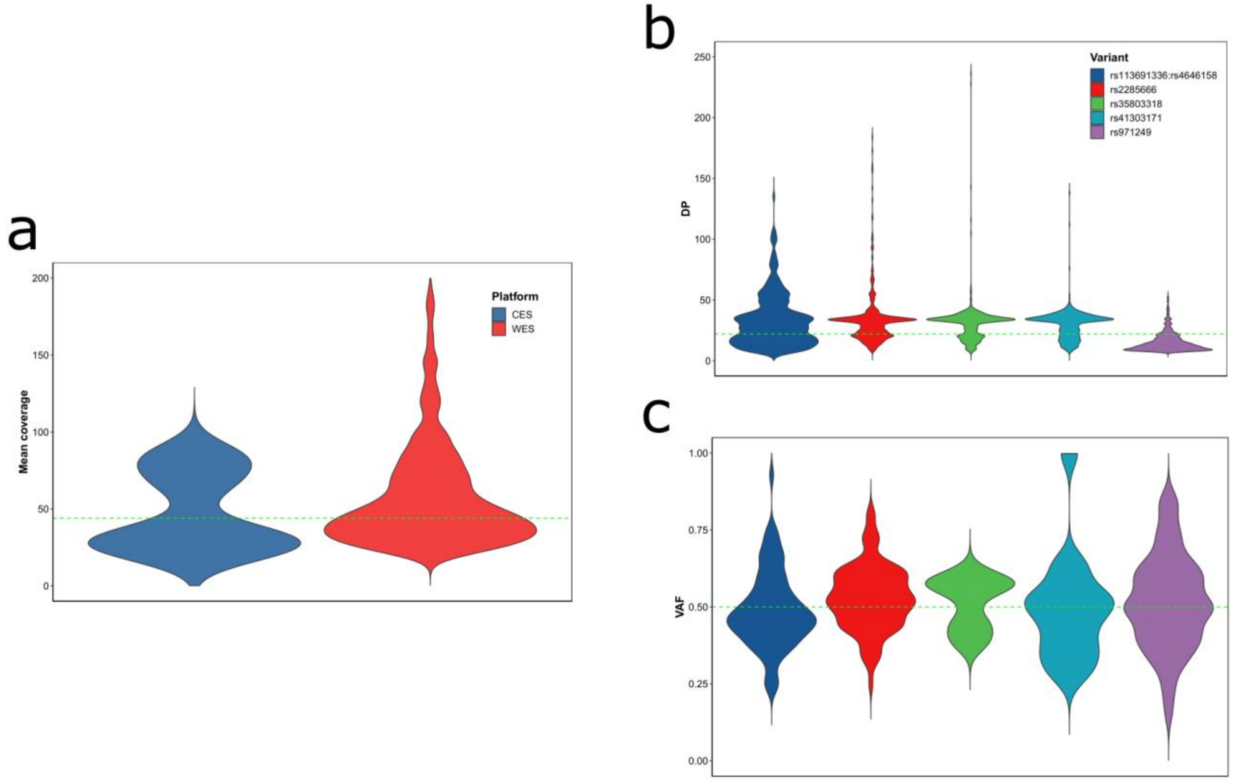

Supplementary Figure S4. Quality control of variants detected in the Russian population. The horizontal greed dashed lines on each plot depict the median values of the parameter. (a). The distribution of mean coverage on target intervals for retained 550 samples. (b). The distribution of site-depth (DP) for 5 functionally significant variants in the Russian population (c). The distribution of variant allele depth (VAF) for 5 functionally significant variants in the Russian population.
